# Cross-Domain Generalization for LiDAR-Based 3D Object Detection in Infrastructure and Vehicle Environments

**DOI:** 10.3390/s25030767

**Published:** 2025-01-27

**Authors:** Peng Zhi, Longhao Jiang, Xiao Yang, Xingzheng Wang, Hung-Wei Li, Qingguo Zhou, Kuan-Ching Li, Mirjana Ivanović

**Affiliations:** 1 School of Information Science and Engineering, Lanzhou University, Lanzhou 730000, China; 2 Department of Computer Science and Information Engineering, Providence University, Taichung 43301, Taiwan; 3 Faculty of Sciences, University of Novi Sad, 21000 Novi Sad, Serbia

**Keywords:** 3D object detection, V2X cooperative perception, LiDAR point clouds, infrastructure sensors

## Abstract

In the intelligent transportation field, the Internet of Things (IoT) is commonly applied using 3D object detection as a crucial part of Vehicle-to-Everything (V2X) cooperative perception. However, challenges arise from discrepancies in sensor configurations between vehicles and infrastructure, leading to variations in the scale and heterogeneity of point clouds. To address the performance differences caused by the generalization problem of 3D object detection models with heterogeneous LiDAR point clouds, we propose the Dual-Channel Generalization Neural Network (DCGNN), which incorporates a novel data-level downsampling and calibration module along with a cross-perspective Squeeze-and-Excitation attention mechanism for improved feature fusion. Experimental results using the DAIR-V2X dataset indicate that DCGNN outperforms detectors trained on single datasets, demonstrating significant improvements over selected baseline models.

## 1. Introduction

The rapid development of Internet of Things (IoT) technology has enabled seamless connectivity between people, vehicles, and transportation infrastructure. Vehicle-to-Everything (V2X) communication not only advances autonomous driving from standalone intelligence to connected intelligent driving but also promotes the development of Intelligent Transportation Systems (ITSs) by enhancing road safety, efficiency, and automation [1,2].

Collaborative perception in vehicular networks is a critical component of ITSs [3], where vehicles share real-time information with infrastructure, other vehicles, and pedestrians to achieve a comprehensive understanding of the environment [4]. This collaborative approach effectively addresses the limitations of autonomous vehicles, such as occlusions, limited viewpoints, and weak long-range perception capabilities. As the core of this process, 3D object detection uses sensors like LiDAR to collect point clouds for recognizing and localizing surrounding objects (e.g., vehicles, pedestrians, and obstacles) [5], which include intermediate features and object bounding boxes, serving as crucial input for multi-agent collaboration.

Nevertheless, existing studies often make an overly simplistic assumption that all collaborating agents use identical sensors and run the same perception models [6]. In real-world scenarios, collaborating agents are likely to be heterogeneous, presenting significant challenges in integrating data from different sources [7], such as roadside and onboard sensors. These sensors typically differ in configuration, positioning, and viewpoints in real-world scenarios, leading to disparities in the generated LiDAR point clouds in density, resolution, and scale. Conventional 3D object detection models, typically trained on homogeneous data, struggle to adapt to such heterogeneity, resulting in degraded detection performance. Therefore, there is an urgent need for a model that can maintain high detection accuracy and exhibit strong generalization capabilities under heterogeneous sensor configurations, particularly between vehicle- and infrastructure-based sensors.

To evaluate these issues, we used the high-fidelity simulator CARLA [8] to create a perception dataset, explicitly considering the impact of viewpoint differences between vehicles and infrastructure, as well as the effect of LiDAR channel count, on model performance, conducting multiple sets of experiments. Figure 3 shows the independent and cross-test performance of the same model under different viewpoints and point cloud densities. The experiments indicate that viewpoint differences between vehicles and infrastructure, and even differences in LiDAR channel count between vehicles, can significantly impact a model’s generalization ability, especially in detecting hard-to-detect objects within the scene, where performance degradation is particularly evident.

Therefore, to address the cross-domain generalization issue caused by the differences between vehicle-mounted and roadside LiDAR point cloud data in vehicular communication systems, this paper proposes a Dual-Channel Generalization Neural Network (DCGNN). The model introduces data calibration operations and receptive field enhancement modules in the data and feature layers, respectively. Experimental results on the DAIR-V2X dataset [9] demonstrate that the DCGNN performs excellently in detection, significantly improving the robustness and accuracy of 3D object detection models across different sensor configurations and complex scenarios.

Due to the current scarcity of infrastructure data, fine-tuning models specifically on this data remains a challenge. In heterogeneous sensor configurations, such as those found in vehicle-to-infrastructure environments, the point cloud data collected from vehicles and infrastructure often exhibit significant differences in distribution, format, and quality. These disparities make it difficult to develop detection models that generalize well across diverse domains. The proposed DCGNN model is designed to address these cross-domain differences by leveraging a two-layer design at both the data level and the network level, thereby enhancing detection robustness across various sensor configurations. By focusing on cross-domain generalization, the DCGNN minimizes the reliance on data-intensive fine-tuning and enables more efficient, real-time processing in complex, collaborative scenarios, providing a novel solution to the domain generalization challenges faced by perception models.

The key contributions of this paper can be summarized as follows:

(1) A 3D object detection model designed explicitly for V2X perception using roadside LiDAR point clouds is introduced to effectively address performance disparities caused by point cloud heterogeneity at both data and feature levels.
(2) A novel data-level downsampling and calibration module, named Voxel-Based Weighted Centroid Downsampling (VWCD), is designed to enable adaptive information fusion between heterogeneous agents.
(3) A novel Cross-Perspective Feature Synergization Module (CPSyM) that leverages both range-view and bird’s-eye-view perspectives is proposed, using a Squeeze-and-Excitation (SE) attention mechanism and Atrous Spatial Pyramid Pooling (ASPP) to fuse multi-view features and enhance the performance of 3D object detectors.

The remainder of this article is organized as follows. Section 2 provides a discussion of related works. Section 3 discusses the differences between roadside and vehicle-mounted LiDAR point clouds. The proposed methodology is described in Section 4, while the experimental results of evaluating object detection performance for both roadside and vehicle-mounted sensors are presented in Section 5. Finally, concluding remarks and directions for future research are given in Section 6.

## 2. Related Works

### 2.1. LiDAR-Based 3D Object Detection

LiDAR-based 3D object detection methods can be categorized into four main types. First, point-based methods directly extract features from raw point clouds. For example, PointNet [10] encodes point cloud features using Multilayer Perceptrons (MLPs), but it struggles to capture local relationships between points. PointNet++ [11] addresses this limitation by enhancing local feature extraction, becoming a foundational approach for many subsequent models. The single-stage detector 3DSSD [12] improves detection speed by removing the Feature Propagation (FP) layer and bounding box refinement. Pointformer [13] incorporates the Transformer architecture, combining local and global features for improved context modeling and detection performance.

Second, voxel-based methods encode features by dividing point clouds into evenly spaced voxel grids. For example, VoxelNet [14] introduced voxel feature encoding (VFE), improving feature representation but with low processing efficiency. SECOND [15] introduces sparse convolutions to avoid redundant computations on empty voxels, significantly boosting speed and establishing itself as a mainstream 3D detection method. PointPillars [16] further enhances detection speed by using cylindrical partitioning, avoiding segmentation along the z-axis. CenterPoint [17] integrates keypoint detection to enhance rotational invariance. Voxel Transformer [18] accelerates voxel querying and strengthens context modeling using sparse voxel modules and attention mechanisms.

Third, projection-based methods detect objects by projecting point clouds into 2D views. RangeRCNN [19] addresses scale and occlusion issues in the range view (RV) using dilated convolutions and the RV-PV-BEV module, while RangeDet [20] optimizes detection with a range-conditioned pyramid and novel convolution operations. Bird’s-eye view (BEV) methods, such as BirdNet [21], project point clouds onto the BEV plane, applying post-processing to generate 3D bounding boxes. BirdNet+ [22] further improves detection performance. Complex-YOLO [23] extends YOLOv2 [24] to 3D detection for efficient object detection.

Finally, graph-based methods use graph neural networks (GNNs) to capture geometric information within point clouds [25]. PointRGCN [26] generates candidate boxes and aggregates point features within those boxes using a residual graph convolutional network (R-GCN), followed by contextual information aggregation using a contextual GCN (C-GCN). Despite its strong performance, this approach has high computational costs. Point-GNN [27] constructs graphs using voxel downsampling, introduces an automatic registration mechanism for enhanced translation invariance, and incorporates bounding box merging and scoring Non-Maximum Suppression (NMS) to improve localization accuracy. PC-RGNN [28] incorporates a multi-scale attention mechanism to aggregate multi-scale graph contextual information, strengthening geometric relationship modeling between points. Graph R-CNN [29] handles uneven point cloud densities using dynamic farthest voxel sampling and region of interest (RoI) graph pooling, combining image features to improve context modeling and detection precision.

In summary, LiDAR-based 3D object detection methods employ a variety of strategies, each with distinct advantages. Point-based techniques excel at direct feature extraction, while voxel-based methods enhance processing speed through structured data. Projection and graph-based approaches improve contextual understanding but may require trade-offs in computational efficiency. Collectively, these methodologies advance the boundaries of accuracy and efficiency in practical applications. Considering the balance between speed and accuracy, PointPillars [16] was selected as the baseline for this study.

### 2.2. V2X Perception and Roadside Perception

In collaborative perception scenarios, intelligent connected vehicles can acquire perception data from surrounding vehicle sensors or roadside devices. This information may be in the form of raw images, point cloud data, or abstract features extracted by perception algorithms. By leveraging multi-view perception data from neighboring intelligent vehicles or roadside infrastructure, it is possible to mitigate the blind spots of the current vehicle’s sensors. For instance, the overhead view from roadside equipment at intersections can reveal occluded vehicles or pedestrians, while the perspective from another vehicle around a corner can expose blind spots that the current vehicle cannot detect.

From the perspective of collaboration timing, V2X collaboration strategies can be classified into early-stage [30,31], mid-stage [6,32,33,34,35,36,37], and late-stage [38,39,40] collaboration. Early-stage collaboration transmits raw data, with subsequent processing occurring on the current vehicle’s system. While this approach offers the highest perception accuracy, it requires the most computational power. Mid-stage collaboration transmits extracted features to the vehicle for fusion and prediction, reducing the computational load at the cost of some loss in perception accuracy. Late-stage collaboration fuses the perception results from multiple sources, with the final processing occurring on the vehicle. Although this approach requires the least computational effort and is less accurate than early-stage or mid-stage collaboration, it still surpasses single-vehicle perception in effectiveness.

Most existing research has focused on vehicle-to-vehicle collaboration, often conducted in idealized experimental conditions, overlooking sensor heterogeneity and variability. In vehicle-to-infrastructure (V2I) collaboration, roadside sensors offer significant advantages due to their higher precision and broader field of view, yet these have not been fully exploited in many studies. Although BEVHeight [41] has explored this issue, its work primarily focuses on visual perception, and research on roadside LiDAR-based point cloud perception remains scarce. To address this gap, we propose a solution to improve the generalization capabilities of 3D object detection models when dealing with heterogeneous LiDAR point cloud data.

## 3. Problem Statement

**Figure 1:**
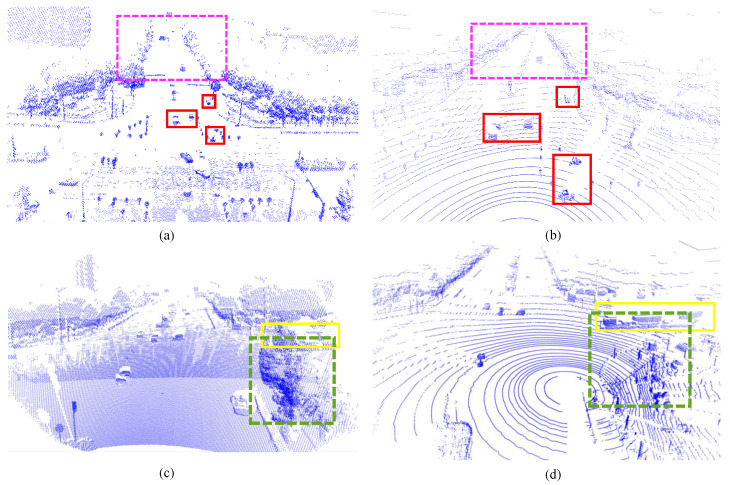
Differences between vehicle-mounted and roadside LiDAR point clouds: (**a**,**c**) show the point cloud from the infrastructure-side LiDAR, and (**b**,**d**) show the point cloud from the vehicle-side LiDAR.

Unlike mobile vehicle-side LiDAR, roadside equipment, such as traffic intersections, is typically installed at fixed positions. As shown in Figure 1, the fixed installation angle and position lead to several differences in the point cloud data compared to vehicle-side LiDAR:

(1) Occlusions: Roadside LiDAR, positioned at a higher vantage point, is more prone to being obstructed by fixed objects such as buildings and trees. For instance, these structures may block roadside sensors at an intersection, limiting their ability to scan objects around corners and resulting in missing point cloud data in certain areas. As shown in Figure 1c,d, the green boxes in both the vehicle-side and roadside views illustrate that objects like trees do not obstruct the vehicle in the green box from the vehicle’s perspective, as they do in the roadside view. Consequently, the vehicle in the yellow box remains undetected due to occlusion by surrounding objects.
(2) Viewpoint, Position, and Point Cloud Density: Vehicle-mounted LiDAR captures more detailed road-level information, as it is closer to the center of the road or driving path. In contrast, roadside LiDAR, positioned at a higher vantage point, provides a broader field of view, allowing it to capture more significant scenes. However, roadside LiDAR tends to produce sparser point clouds, especially at greater distances, where the density decreases even with additional laser beams. As a result, while roadside LiDAR offers a broader perspective, point cloud data are often less detailed and less dense than vehicle-mounted LiDAR, particularly in distant regions. As shown in Figure 1a,b, the target outlines captured by the roadside LiDAR are less distinct and can easily be confused with surrounding objects.
(3) Environmental Conditions and Reflectivity: Roadside LiDAR, with its broader coverage, is more vulnerable to changes in ambient light, weather conditions, and the reflectivity of objects, as shown in the pink boxes in Figure 1a,b. These factors can adversely affect the quality of the point cloud data in specific regions.

Currently, most perception models, such as those for object detection and semantic segmentation, are designed and trained using vehicle-mounted LiDAR point clouds. When applied to V2X scenarios, the models often fail to account for the differences between vehicle and roadside LiDAR data. While vehicle-side predictions remain accurate, performance drops sharply when these models are employed for roadside data.

**Figure 2:**
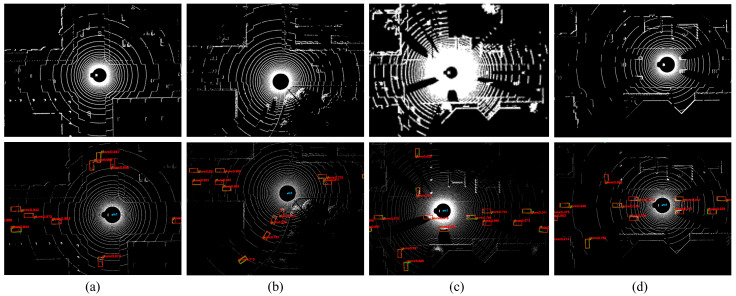
Comparison of LiDAR channel count and spatial angle point cloud visualizations: (**a**) Vehicle-mounted 32-channel LiDAR point cloud; (**b**) Roadside 32-channel LiDAR point cloud; (**c**) Vehicle-mounted 128-channel LiDAR point cloud; (**d**) Vehicle-mounted 32-channel LiDAR point cloud. It is noteworthy that the point clouds in (**a**,**b**) were recorded in the same scene, while the point clouds in (**c**,**d**) were recorded in the same scene.

**Figure 3:**
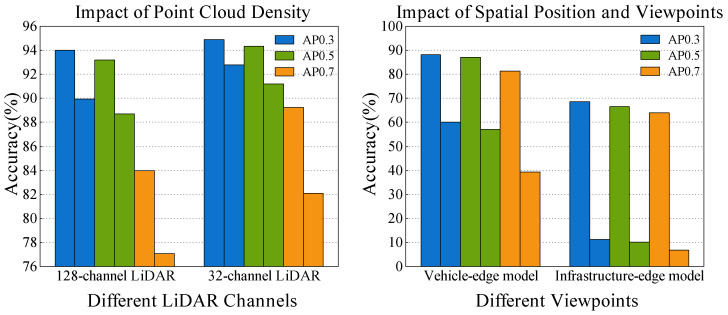
We evaluated the performance of the PointPillars model under different scenarios, focusing on variations in metrics such as AP0.3, AP0.5, and AP0.7. The left figure illustrates the inference results of models trained on point clouds from 128-channel and 32-channel LiDARs, evaluated through independent testing on their respective test sets and cross-testing on test sets from the other configuration. The right figure shows the inference outcomes of models trained using vehicle-mounted and infrastructure-mounted LiDAR data, subjected to independent testing on their respective datasets and cross-testing on datasets from the opposite perspective.

Based on the second point above regarding the influence of viewpoint, position, and point cloud density, we constructed multiple datasets under various traffic scenarios within the CARLA environment, as shown in Figure 2. These datasets include both perception data from vehicles and roadside units with the same LiDAR channel count in the same scenario, as well as perception data from the same scenario with different LiDAR channel counts. We divided the two perception datasets into two groups based on the LiDAR channel count and spatial viewpoint, as shown in Figure 2a,b, and Figure 2c,d, and conducted cross-testing and independent testing. The results shown in Figure 3 demonstrate that differences in LiDAR channel count and spatial viewpoint both affect model performance, especially in detecting challenging cases, where performance degradation is particularly pronounced.

To address this issue, we propose the DCGNN, which introduces data correction operations and a receptive field enhancement mechanism at both the data and feature levels, enabling the perception model to maintain high detection accuracy and strong generalization capabilities under heterogeneous sensor configurations.

## 4. Method

We first provide a comprehensive overview of the proposed framework, followed by an in-depth discussion of the Voxel-Based Weighted Centroid Downsampling (VWCD) module. Then, we elaborate on the Cross-Perspective Feature Synergization Module (CPSyM).

### 4.1. Overall Architecture

**Figure 4:**
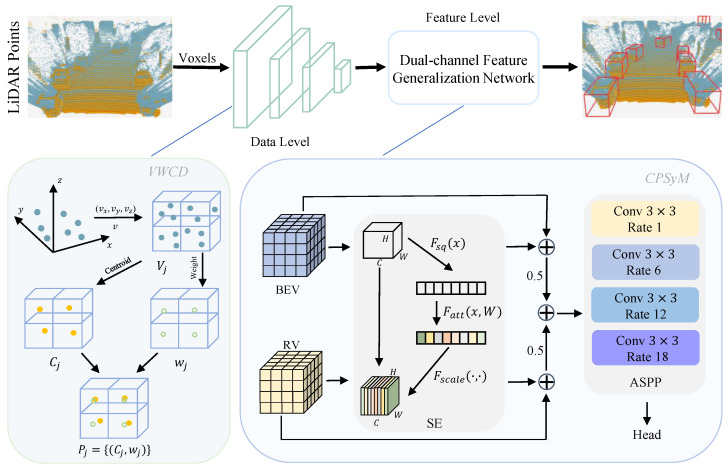
The structure of the DCGNN: The bottom-left part of the figure depicts the VWCD module, and the bottom-right part shows the CPSyM.

As illustrated in Figure 4, the proposed DCGNN framework consists of two main stages. The first stage, the VWCD module, involves data-level downsampling and calibration. Assuming that the initial point cloud consists of points with coordinates $\left(x_{i},y_{i},z_{i}\right)$, the entire point cloud space is divided into a uniform 3D voxel grid based on a predefined voxel size. For each voxel, a weighted centroid representing the voxel is calculated using all the points within that voxel. This centroid is used in subsequent processing instead of all the points within the voxel. Additionally, a representative weight for the voxel is computed, indicating its overall importance. Thus, the downsampled point cloud consists of these weighted centroids and their corresponding weights, significantly reducing the size of the point cloud while preserving key structural information and characteristics.

The second stage, the CPSyM, focuses on feature extraction at the network level. The downsampled point cloud is first processed by an encoder to extract the initial features, which are then passed through the backbone network for spatial feature extraction. Following this, the spatial features pass through an intermediate convolutional layer and are directed to separate BEV and RV layers to generate BEV and RV feature representations. The CPSyM first applies the Squeeze-and-Excitation (SE) attention mechanism to the BEV and RV features individually to calculate attention scores for each. Subsequently, these attention scores are added to their respective original features, and the enhanced BEV and RV features are each weighted by 0.5 to achieve balanced fusion. The weighted BEV and RV features are then summed to generate an aggregated feature representation.

This aggregated feature representation is then fed into the Atrous Spatial Pyramid Pooling (ASPP) module, which applies multi-scale convolutions with dilation rates of 1, 6, 12, and 18 to further extract contextual information. By employing atrous convolutions with different dilation rates, the ASPP module extends the receptive field, combining both fine-grained and wide-range contextual information to enhance multi-scale feature representation. The output features from the ASPP module are finally passed to the network’s detection head to produce the final detection results. This design fully integrates multi-view information from BEV and RV, and through multi-scale feature capture, improves the model’s adaptability and detection accuracy in complex scenes.

Finally, the output of the CPSyM is fed into the detection head to produce the final detection results.

### 4.2. VWCD

To address the differences in point cloud density and detail, as well as issues such as environmental conditions and occlusions, as described in Section 3, we propose the DCGNN incorporating the VWCD module. This approach effectively reduces the number of points while retaining critical information from the point cloud, offering improved denoising and flexibility. The VWCD module achieves this through point density consideration, weighted centroid computation, and dynamic adjustment of retained points. The algorithmic process is described below.

First, we fix the voxel size and partition the point cloud into different spaces. The voxel size affects the downsampling outcome, and in the DCGNN, it is set according to the DAIR-V2X LiDAR’s beam width and range, with a value of *v*. The process is described in Algorithm 1.
**Algorithm 1** Voxel partitioning of a point cloud**Require:** Point cloud with coordinates $\left(x_{i},y_{i},z_{i}\right)$, voxel size *v***Ensure:** Voxel indices $v_{x},v_{y},v_{z}$ for each point 1:**for** each point $\left(x_{i},y_{i},z_{i}\right)$ in the point cloud **do** 2:      $v_{x}\leftarrow \left\lfloor \frac{x_{i}}{v}\right\rfloor$ 3:      $v_{y}\leftarrow \left\lfloor \frac{y_{i}}{v}\right\rfloor$ 4:      $v_{z}\leftarrow \left\lfloor \frac{z_{i}}{v}\right\rfloor$ 5:      Process voxel indices $v_{x},v_{y},v_{z}$ 6:**end for**

After partitioning the point cloud into voxels, we compute the weighted centroid for the points within each voxel. Traditional voxel sampling methods often rely on random selection or central point strategies, which can lead to a suboptimal representation of the original geometric structure. To improve the representativeness of the downsampled points, we calculate a more precise weighted centroid by assigning weights to each point, as shown in Equation (1). This approach ensures that the downsampled points preserve the geometric features of the original point cloud more accurately.

(1) $$C_{j}=\left(\frac{\sum_{i=1}^{N}w_{i}x_{i}}{\sum_{i=1}^{N}w_{i}},\frac{\sum_{i=1}^{N}w_{i}y_{i}}{\sum_{i=1}^{N}w_{i}},\frac{\sum_{i=1}^{N}w_{i}z_{i}}{\sum_{i=1}^{N}w_{i}}\right)$$

In Equation (1), $C_{j}$ represents the weighted centroid of voxel *j*, where $w_{i}$ is the weight of the *i*-th point within the voxel and $\left(x_{i},y_{i},z_{i}\right)$ are the coordinates of that point. The summation runs over all *N* points in the voxel, ensuring that points with higher significance (higher weights) contribute more to the final centroid position.

As a space-partitioning algorithm, voxel sampling offers low time complexity but can result in uneven point distribution, especially in outdoor scenarios. Fixed voxel methods often lead to significant discrepancies in the number of sampled points between dense and sparse regions, causing already sparse distant point clouds to become even sparser. This can negatively impact the accuracy and recall of distant object detection, ultimately affecting the overall model precision.

To address this issue, we incorporate weight calculations, as shown in Equation (2). By using weighted averaging, we ensure that points with significant features have a greater influence on the final representative points of the voxel, thereby enhancing the representation of key points. For example, in dense regions, despite the reduction in point count, the weighted centroid and weight adjustments help retain more critical information, preventing the excessive simplification of features in dense areas due to downsampling.

(2) $$w_{j}=\frac{\sum_{i=1}^{N}w_{i}^{2}}{\sum_{i=1}^{N}w_{i}}$$

Finally, we combine the weighted centroid $C_{j}$ and the computed weight $w_{j}$ to produce the downsampled point cloud output, as shown in Equation (3):

(3) $$P_{j}=\left\{C_{j},w_{j}\right\}|\forall j\in \mathrm{voxels}$$

This process results in the final downsampled and corrected point cloud data for subsequent processing.

### 4.3. CPSyM

To address the differences between roadside and vehicle-mounted LiDAR point clouds, we propose the CPSyM, inspired by the VISTA model [42] and the method in [43]. This module leverages multi-view feature inputs and employs a balanced fusion approach to ensure more accurate and robust feature representation for roadside LiDAR point clouds. The balanced fusion strategy integrates features from BEV (bird’s-eye view) and RV (range view) perspectives, allowing complementary information from both views to be optimally utilized. BEV provides rich spatial context, which is essential for understanding the global layout, while RV captures fine-grained geometric details, critical for detecting small or occluded objects. This balanced fusion ensures that both feature sets contribute effectively, thus enhancing model performance and generalization capability.

Firstly, CPSyM utilizes the bird’s-eye-view (BEV) and range view (RV) features as multi-view inputs. These two perspectives can capture rich scene information from different geometric spaces. For the BEV and RV features generated by the model, we apply the SE attention mechanism separately. The SE module computes channel-level attention distributions for each view feature, capturing the global spatial response weights. Specifically, the SE module applies global average pooling to aggregate the BEV and RV features, respectively, followed by a series of linear transformations and activation functions to adaptively adjust the weights of each channel. For each view feature $X\in R^{C\times H\times W}$, channel attention is calculated using the following equation:

(4) $$z=\sigma (W_{2}\cdot \delta \left(W_{1}\cdot s\right))$$

(5) $$s=\frac{1}{H\times W}\sum_{i=1}^{H}\sum_{j=1}^{W}X\left(i,j\right)$$

where $W_{1}$ and $W_{2}$ are learnable weight matrices, δ and σ denote activation functions, and *s* represents the global average pooling vector. Through this process, the BEV and RV features not only retain their structural information but also enhance their responsiveness to critical feature channels.

Once the attention scores for the BEV and RV features are obtained, these scores are added element-wise to their respective original features to generate enhanced view features. To balance the influence of these features during fusion, we apply a weighting factor of 0.5 to the enhanced BEV and RV features individually. This weighting scheme is designed to balance the contribution from each view, avoiding the dominance of a single view and achieving a more robust multi-view feature representation.

Subsequently, the weighted BEV and RV features are summed to obtain the final aggregated feature representation. This aggregated feature representation integrates spatial and channel information from multiple views, exhibiting stronger discriminability and robustness. To further enrich the multi-scale information within this feature representation, the aggregated features are input into the ASPP module, which performs multi-scale feature extraction with different dilation rates. The output feature $Y_{d}$ for each branch in the ASPP module is computed as follows:

(6) $$Y_{d}={\mathrm{Conv}}_{d}\left(X\right)=\sum_{i=-k}^{k}\sum_{j=-k}^{k}w\left(i,j\right)\cdot X\left(x+d\cdot i,y+d\cdot j\right)$$

where ${\mathrm{Conv}}_{d}$ denotes the convolution operation with dilation rate *d*, *k* is the kernel size, and *w* represents the convolution kernel weights.

The ASPP module applies dilated convolutions with dilation rates of 1, 6, 12, and 18 to achieve multi-scale feature extraction and effectively capture diverse contextual information. First, the input feature map passes through an initial convolutional block (pre_conv), implemented as a BasicBlock to enhance feature representation prior to multi-scale fusion. This initial stage optimizes the foundational features, preparing them for multi-scale processing.

The ASPP module then leverages a 1 × 1 convolutional layer to generate a base feature branch (branch 1 × 1), which preserves fine-grained information while reducing computational complexity. The core of the ASPP operation lies in its multi-branch design, wherein we apply dilated convolutions with different dilation rates on separate branches (branch1, branch6, branch12, branch18). This design is implemented using a shared, learnable convolutional kernel (self.weight) that performs dilation-specific convolutions as follows:

1. A dilation rate of 1 yields standard convolution, focusing on local fine-grained details crucial for object recognition.
2. A dilation rate of 6 extends the receptive field to capture mid-range contextual information.
3. Higher dilation rates of 12 and 18 further broaden the receptive field, allowing the module to aggregate spatial information from a broader context and enabling a more comprehensive understanding of the scene.

The multi-scale features extracted from each branch are concatenated along the channel dimension and combined with the original feature map to form a unified multi-scale feature representation. The concatenated features are subsequently processed through a post-fusion convolutional block (post_conv), implemented as a ConvBlock, to perform channel fusion and produce the final, enhanced multi-scale feature representation. This structure combines detailed spatial features with global contextual cues, enhancing the feature representation’s robustness and discriminative power.

To optimize computational efficiency during gradient computation, we employ a checkpoint mechanism that conserves memory by delaying certain intermediate computations, making it particularly suitable for deep network training. The enhanced output from the ASPP module is then forwarded to the network’s head for downstream prediction tasks. This multi-scale ASPP module design significantly augments feature expressiveness, contributing to improved model adaptability and predictive accuracy across complex scenarios.

## 5. Experimental Results

### 5.1. Dataset

The DAIR-V2X dataset is a real-world dataset designed for vehicle–road cooperative perception. Each scenario includes a vehicle and a roadside unit equipped with a LiDAR and a camera. Notably, there are significant differences between the vehicle-side and roadside LiDARs regarding the number of channels and spatial positioning within the scene. The experiments revealed that these differences further impact the model’s generalization performance.

In this work, we focused on the DAIR-V2X-C dataset, which specifically leverages point clouds from roadside-mounted LiDARs to investigate roadside perception. The DAIR-V2X-C dataset contains a total of 38,845 frames of point cloud data, with the roadside using a 300-channel LiDAR and the vehicle using a 40-channel LiDAR. The dataset was split into training, validation, and test sets with a 50%, 20%, and 30% distribution, respectively. The training set consisted of 19,423 frames, the validation set contained 7769 frames, and the test set included 11,653 frames. We trained the model separately using point clouds from both the vehicle side and infrastructure side and evaluated it in a cooperative perception scenario. Detection performance was measured using the average precision (AP) at Intersection-over-Union (IoU) thresholds of 0.3, 0.5, and 0.7. AP evaluates the accuracy of object detection by calculating precision at various recall levels. IoU thresholds of 0.3, 0.5, and 0.7 correspond to the minimum overlap between the predicted and ground-truth boxes required for a detection to be considered correct. AP@0.3 is more lenient, with a 30% overlap; AP@0.5 is the standard, requiring 50%; and AP@0.7 is stricter, demanding 70% overlap for a detection to be deemed accurate.

Additionally, we generated heterogeneous vehicle and infrastructure data using the CARLA simulator. Specifically, we created five distinct scenarios, each involving both 128-line and 32-line LiDAR sensors. The training set included 4984 samples from the vehicle and 4984 samples from the infrastructure, totaling 9968 samples. The test set contained 803 samples from the vehicle and 803 samples from the infrastructure, totaling 1606 samples.

### 5.2. Settings

**Model Configuration:** In the data preprocessing stage, point clouds were divided into pillars, with both vehicle-side and roadside point cloud pillars set to a size of [0.4m,0.4m]. We employed BEV and RV feature extractors to generate 2D feature maps for BEV and RV perspectives. These single-view features were then passed through a general fusion channel commonly used in existing fusion algorithms for feature integration. The fused features were subsequently fed into the detection head for object detection.

**Experiment Setup:** To evaluate the model’s performance under different data conditions, we conducted two main experiments on the DAIR-V2X-C dataset using vehicle-side and infrastructure-side data. The model was trained on a single data source and tested on cooperative point cloud data for inference and detection. The same experimental settings were adopted as in the baseline methods, and comparative and ablation experiments were performed to thoroughly verify our proposed modules’ effectiveness.

**Training Details:** The model was trained using the Adam optimizer with a one-cycle learning rate strategy. The initial learning rate was set to 0.002, and a weight decay of $10^{-4}$ was applied. Training was conducted over 20 epochs. The experiments were performed on a system equipped with an Intel i7-11700K CPU, an NVIDIA RTX 3090 Ti GPU, and 32 GB of RAM. The system ran on Ubuntu 20.04 LTS, with CUDA 11.1 to accelerate GPU computation. All models were implemented using PyTorch 1.10.1 within a conda environment configured with Python 3.8.19. Common data augmentation techniques were applied during training, including random scene flipping, random scaling, random rotation, and translation. The detection range for the dataset was set to [−140.8m,140.8m] horizontally and [−3m,1m] vertically, and the base voxel size was set as [0.4m,0.4m,4m]. During inference, we employed the NMS strategy with the threshold set to 0.15.

### 5.3. Performance Evaluation and Comparison

To evaluate the performance of the DCGNN under different data conditions, we first conducted baseline experiments on the vehicle-side and infrastructure-side point clouds from the DAIR-V2X-C dataset. In these baseline experiments, the model was trained solely on either vehicle-side or infrastructure-side data, and the trained models were then deployed for inference on the DAIR-V2X-C dataset.

Building on this, we introduced the VWCD module for downsampling roadside point clouds to achieve a point density similar to that of the vehicle-side point clouds. Additionally, we optimized the network structure by incorporating the CPSyM. The VWCD module downsampled the roadside point clouds to match the density of the vehicle-side point clouds, while the CPSyM fused multi-view features to enhance the model’s ability to perceive point cloud features. The improved models were trained separately on the vehicle-side and infrastructure-side datasets and then deployed for inference on the DAIR-V2X-C dataset to generate the detection results.

The experimental results presented in Table 1 indicate that, after applying point cloud downsampling and optimizing the network structure, the detection accuracy of the improved vehicle-side model increased by 0.7% in AP@0.7 on the DAIR-V2X-C dataset compared to the baseline model, while the detection accuracy of the enhanced infrastructure-side model increased by 0.5% in AP@0.7. These results validate the effectiveness of integrating the VWCD module and CPSyM in enhancing detection performance, particularly in multi-source data fusion scenarios for vehicle–road cooperation, where the improved model more accurately captures target information and enhances detection accuracy.

**Figure 5:**
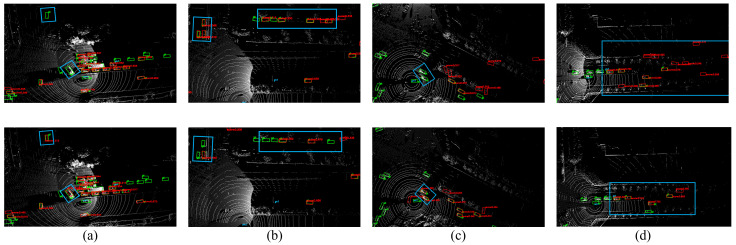
Comparison of detection performance between the baseline model and the improved model: the first row is the baseline model, and the second row is the improved model; (**a**,**b**) are the vehicle-side model, and (**c**,**d**) are the infrastructure-side model.

Figure 5 illustrates a visual comparison between our improved model and the baseline model in the vehicle detection task. Specifically, Figure 5a,b compare the vehicle-side model’s performance on the cooperative dataset, highlighting the advantages of the improved model.

In Figure 5a, the improved model successfully detects distant vehicles that the baseline model fails to capture. Distant vehicles are typically difficult to detect due to the low density of the point clouds and indistinct target features, which poses challenges for the baseline model. However, our proposed model, through the enhanced point cloud preprocessing and feature extraction modules, increases its ability to detect hidden targets in sparse point clouds, resulting in better performance in long-range scenarios and allowing the improved model to effectively capture distant vehicles, particularly those that are large but have blurry features. Additionally, the improved model detects vehicles adjacent to the ego vehicle, crucial for enhancing the perception of the vehicle’s surrounding environment. This capability is essential in dynamic driving environments, improving safety and response times.

Figure 5b further demonstrates the improved model’s significant enhancements in detecting parked vehicles along the roadside and vehicles near intersections or corners. The baseline model struggles in these complex scenarios, where occlusions and a limited field of view can lead to missed or incorrect detections. By optimizing geometric feature extraction, our improved model strengthens its ability to capture and understand targets in complex environments. Particularly in corner cases or situations with heavy occlusion, the improved model can better identify parked vehicles or those hidden at the edges of the sensor’s field of view, showcasing its robustness across multi-scene and multi-angle conditions.

Figure 5c,d compare the infrastructure-side model’s performance, further demonstrating the superiority of the improved model. Figure 5c shows that the enhanced model can more accurately detect vehicles near the roadside sensor, significantly improving the perception of vehicles close to the ego vehicle. This is crucial for strengthening the collaborative perception between the vehicle and infrastructure-side equipment, effectively reducing blind spots and increasing the accuracy of road safety and information sharing. Figure 5d shows that the improved model significantly reduces false positives. The baseline model is prone to false detections in complex traffic environments, leading to unnecessary alerts or incorrect actions. The enhanced model, through more refined feature extraction and classification mechanisms, effectively reduces false positives, improving the reliability of the detection results.

Overall, these comparative results fully demonstrate the advantages of our method. The improved model exhibits strong robustness and superior performance in vehicle–road collaborative perception scenarios by accurately detecting vehicles in distant, nearby, and complex scenarios. It significantly improves detection accuracy while greatly reducing false detection rates. These improvements are critical for real-world applications, as they enhance vehicle safety and collaborative perception, further validating the effectiveness and reliability of our method.

**Figure 6:**
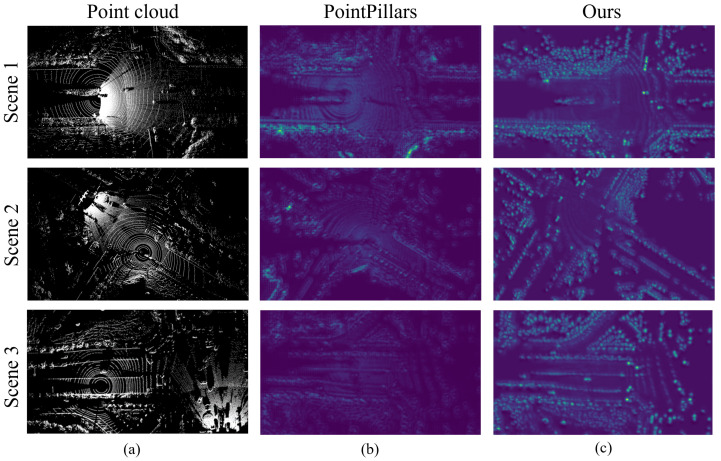
Comparison of feature extraction performance between the baseline model and our improved model: (**a**) original point cloud visualization, (**b**) baseline model (PointPillars), and (**c**) improved model; The rows represent different scenes (Scene 1, Scene 2, and Scene 3).

Figure 6 illustrates a visual comparison between the original point cloud, the baseline model (PointPillars), and our improved model in processing point cloud data. Specifically, the figure highlights three different scenes and evaluates the models’ ability to extract and represent features effectively in their respective heatmaps.

In Figure 6a, the original point cloud visualizations represent the raw data input to the models, showcasing the complex spatial structures and varying densities across different scenes. These visualizations emphasize the challenges posed by sparse regions and intricate environmental layouts, which require advanced feature extraction techniques to process effectively.

Figure 6b shows the heatmaps generated by the baseline PointPillars model. While the baseline model captures the general structure of the scene, it exhibits limitations in distinguishing critical features, especially in sparse or occluded areas. For instance, in Scene 1, the baseline model struggles to emphasize the boundaries of key obstacles and fails to capture sufficient feature intensity in critical regions, such as intersections or areas with overlapping objects. Similar shortcomings are observed in Scenes 2 and 3, where the feature activations are diffused, and the boundaries of important objects are poorly defined. These limitations stem from the baseline model’s reduced capability to adapt to varying point cloud densities and its reliance on global feature extraction methods.

Figure 6c shows the heatmaps produced by our improved model. In comparison to the baseline model, the improved model demonstrates superior performance in capturing fine-grained features and enhancing critical regions within the scene. For example, in Scene 1, the improved model highlights the boundaries of roads and obstacles with greater clarity and precision, which is essential for downstream tasks such as object detection or semantic segmentation. Similarly, in Scenes 2 and 3, our model successfully amplifies features in sparse regions and complex geometries, such as intersections and areas with overlapping objects. These improvements can be attributed to the enhanced point cloud preprocessing and optimized feature extraction modules, which adaptively focus on key spatial structures while maintaining robust generalization across varying scenarios.

Additionally, the improved model exhibits greater robustness in identifying critical features in challenging environments. For instance, in Scene 3, where dense structures and occlusions dominate the scene, our model maintains strong feature intensity and clear activations, enabling a more accurate representation of the scene’s geometry. This highlights the model’s capability to handle occlusions and complex environmental layouts effectively.

Overall, the comparative analysis of these heatmaps demonstrates the effectiveness of our improved model. By addressing the limitations of the baseline model and focusing on both global and local feature representations, our model enhances the ability to extract meaningful features from point cloud data, especially in sparse or complex scenarios. These improvements are critical for advancing downstream tasks in autonomous driving and collaborative perception, further validating the practical value of our approach.

Furthermore, we conducted a comparative experiment with the PointPillars algorithm on the simulation dataset, which includes both 128-line and 32-line LiDAR sensors. The model was trained on a 3090 Ti platform with a batch size of 8. After training, both independent and cross-validation tests were performed. The experimental results are shown in Table 2.

The above table demonstrates the model’s accuracy on our simulation dataset. Since the data were generated in a simulated environment, the accuracy is naturally higher compared to that on real-world datasets [44]. Across all scenes, our method consistently delivered optimal performance and demonstrated strong generalization capabilities. Additionally, we achieved a frames per second (FPS) rate of 24 on a desktop equipped with an NVIDIA 3090 Ti GPU and an Intel 13700K processor, fully meeting the real-time detection requirements of current LiDAR systems, which typically operate at frame rates ranging from 10 to 20 frames per second.

### 5.4. Ablation Experiments

We conducted ablation experiments to assess the impact of the VWCD and CPSyM components, as presented in Table 3 and Table 4. Table 3 displays the results for the roadside model, while Table 4 presents the vehicle-side model’s outcomes. The results demonstrate that these components significantly contribute to enhancing collaborative performance. Specifically, we evaluated the effects of different combinations of the VWCD, SE, and ASPP modules on the model’s accuracy (mAP), highlighting the individual contributions of each component to the overall performance improvements.

When no additional modules were incorporated, the model’s baseline detection accuracy remained relatively low. However, the addition of the SE or ASPP module individually resulted in a 1–2% improvement in detection accuracy. Specifically, the SE module enhanced the feature representation through its attention mechanism, while the ASPP module improved the model’s adaptability to targets of varying scales via multi-scale feature extraction. Moreover, the standalone application of the VWCD module led to a notable improvement, boosting detection accuracy by approximately 2–4%. Within our framework, the VWCD module played a crucial role in maintaining feature consistency at the data level, effectively mitigating the detrimental effects of data heterogeneity on model performance.

In the experiments involving combinations of modules, all pairwise combinations resulted in substantial improvements in detection accuracy. Notably, the combination of the VWCD and ASPP modules achieved the most significant performance gain, followed closely by the combination of the VWCD and SE modules. This further underscores the synergistic effect of combining data consistency with enhanced feature representation. These results emphasize the importance of addressing data heterogeneity in improving model performance. Additionally, the combination of the SE and ASPP modules also improved detection accuracy, albeit to a slightly lesser extent than the other two combinations.

When the VWCD, SE, and ASPP modules were used simultaneously, the model achieved its highest detection accuracy, with an average improvement of approximately 5–6% over the baseline. This validates the synergistic effect of these three modules, which effectively enhanced data consistency, feature attention, and multi-scale feature representation, thereby maximizing the model’s detection capability in infrastructure-side scenarios.

In contrast, the ablation results for the vehicle-side model show that, due to its relatively high baseline detection accuracy (significantly higher than that of the infrastructure-side model), the performance gains from individual modules were smaller but still meaningful. Specifically, adding the VWCD, SE, or ASPP modules individually improved detection accuracy by approximately 0.5–1%. The standalone application of the VWCD module in the vehicle-side model similarly demonstrated strong data consistency. The SE module further improved local and global feature representations in vehicle-side scenarios, while the ASPP module contributed to enhancing the model’s adaptability to targets of various scales.

In experiments involving combinations of modules, the vehicle-side model also exhibited synergistic effects, although to a lesser extent than the infrastructure-side model. For instance, the combination of the SE and ASPP modules resulted in the highest performance gain, followed by the combination of the VWCD and ASPP modules. The combination of the VWCD and SE modules yielded relatively smaller improvements compared to the other two combinations. When all three modules—VWCD, SE, and ASPP—were applied simultaneously, the vehicle-side model achieved its highest detection accuracy, with an average improvement of 1–2% over the baseline. Although the performance gains were less pronounced than those observed in the infrastructure-side model, they still validate the effectiveness of these modules in high-baseline accuracy scenarios.

Overall, the ablation results for both the infrastructure-side and vehicle-side models highlight the critical roles of the VWCD module and the CPSyM in enhancing detection performance. Their combined application further strengthens the system’s overall detection capabilities in vehicle–road cooperative scenarios, underscoring the necessity of robust data preprocessing and model optimization.

## 6. Concluding Remarks

In this work, we present the DCGNN model to tackle the challenges of 3D object detection using heterogeneous LiDAR point clouds from both infrastructure- and vehicle-mounted sensors. The key contribution of this work is the introduction of the CPSyM, which facilitates effective cross-view feature fusion, and the VWCD module, which improves data consistency. Simulation experiments on the DAIR-V2X dataset demonstrate the model’s superior generalization ability and performance compared to traditional methods, achieving significant improvements, particularly in heterogeneous sensor configurations. These findings validate the effectiveness of the proposed modules in bridging domain gaps and enhancing the robustness of collaborative perception systems. Additionally, our ablation studies highlight the individual and combined contributions of the VWCD, SE, and ASPP modules, emphasizing their importance in improving model accuracy across various sensor domains. In future work, we plan to expand the current framework to include intermediate feature fusion, further bridging the domain gap between infrastructure and vehicle-mounted LiDAR sensors. Additionally, efforts will be directed toward optimizing the model for lightweight deployment, enhancing its suitability for real-time applications in intelligent transportation systems.

## Figures and Tables

**Table 1:** Generalization performance comparison of vehicle-side and infrastructure-side models on cooperative point clouds.

Training Set	Model	mAP		
		**AP30**	**AP50**	**AP70**
vehicle-side	PointPillars	65.29	61.68	48.74
	DCGNN	67.15 (↑ 1.86%)	63.19 (↑ 1.51%)	49.21 (↑ 0.47%)
infrastructure-side	PointPillars	15.59	14.85	11.31
	DCGNN	21.16 (↑ 5.57%)	19.72 (↑ 4.87%)	13.85 (↑ 2.54%)

**Table 2:** Generalization performance comparison of vehicle-side and infrastructure-side models on simulation dataset.

Training Set	Testing Set	Model	mAP		
			**AP30**	**AP50**	**AP70**
128-channel LiDAR	128-channel LiDAR	PointPillars	93.71	92.62	84.53
		DCGNN	95.04	93.35	86.45
	32-channel LiDAR	PointPillars	90.93	89.88	80.25
		DCGNN	91.82	91.10	84.13
32-channel LiDAR	128-channel LiDAR	PointPillars	90.92	90.47	83.44
		DCGNN	90.93	91.10	84.13
	32-channel LiDAR	PointPillars	93.02	92.63	88.45
		DCGNN	95.52	94.60	87.30

**Table 3:** The impact of the VWCD, SE, and ASPP modules on the infrastructure-side model’s performance.

Infrastructure-Side			mAP		
**VWCD**	**SE**	**ASPP**	**AP30**	**AP50**	**AP70**
			15.59	14.85	11.31
✓			17.78	16.68	12.14
	✓		16.40	15.58	11.91
		✓	19.49	18.46	13.09
✓	✓		17.39	16.52	12.44
✓		✓	18.16	17.25	12.49
	✓	✓	18.04	17.30	12.80
✓	✓	✓	21.16	19.72	13.85

**Table 4:** The impact of the VWCD, SE, and ASPP modules on the vehicle-side model’s performance.

Vehicle-Side			mAP		
**VWCD**	**SE**	**ASPP**	**AP30**	**AP50**	**AP70**
			65.29	61.68	48.74
✓			65.91	62.27	48.62
	✓		66.48	62.82	48.79
		✓	65.81	62.30	48.44
✓	✓		65.81	62.30	50.20
✓		✓	66.46	62.81	48.80
	✓	✓	67.14	63.17	49.17
✓	✓	✓	67.15	63.19	49.21

## Data Availability

DAIR-V2X, https://air.tsinghua.edu.cn/DAIR-V2X/cheluduan.html, accessed on 24 January 2025.
